# ERP evidence of attentional somatosensory processing and stimulus-response coupling under different hand and arm postures

**DOI:** 10.3389/fnhum.2023.1252686

**Published:** 2023-11-01

**Authors:** Tetsuo Kida, Takeshi Kaneda, Yoshiaki Nishihira

**Affiliations:** ^1^Higher Brain Function Unit, Department of Functioning and Disability, Institute for Developmental Research, Aichi Developmental Disability Center, Kasugai, Japan; ^2^Faculty of Education, Hakuoh University, Oyama, Japan; ^3^Graduate School of Comprehensive Human Sciences, University of Tsukuba, Tsukuba, Japan

**Keywords:** touch, somatosensation, attention, resource, cognition, anatomical space, physical space, P3

## Abstract

We investigated (1) the effects of divided and focused attention on event-related brain potentials (ERPs) elicited by somatosensory stimulation under different response modes, (2) the effects of hand position (closely-placed vs. separated hands) and arm posture (crossed vs. uncrossed forearms) on the attentional modulation of somatosensory ERPs, and (3) changes in the coupling of stimulus- and response-related processes by somatosensory attention using a single-trial analysis of P300 latency and reaction times. Electrocutaneous stimulation was presented randomly to the thumb or middle finger of the left or right hand at random interstimulus intervals (700–900 ms). Subjects attended unilaterally or bilaterally to stimuli in order to detect target stimuli by a motor response or counting. The effects of unilaterally-focused attention were also tested under different hand and arm positions. The amplitude of N140 in the divided attention condition was intermediate between unilaterally attended and unattended stimuli in the unilaterally-focused attention condition in both the mental counting and motor response tasks. Attended infrequent (target) stimuli elicited greater P300 in the unilaterally attention condition than in the divided attention condition. P300 latency was longer in the divided attention condition than in the unilaterally-focused attention condition in the motor response task, but remained unchanged in the counting task. Closely locating the hands had no impact, whereas crossing the forearms decreased the attentional enhancement in N140 amplitude. In contrast, these two manipulations uniformly decreased P300 amplitude and increased P300 latency. The correlation between single-trial P300 latency and RT was decreased by crossed forearms, but not by divided attention or closely-placed hands. Therefore, the present results indicate that focused and divided attention differently affected middle latency and late processing, and that hand position and arm posture also differently affected attentional processes and stimulus–response coupling.

## Introduction

Humans often face situations that require attention to both hands, such as typing, driving, cooking, playing sports, and playing an instrument. In some of these activities, the focus of attention is sometimes directed to the body part under different positions, such as crossing the forearms and placing the hands closely or separately, with and without overt motor behavior. Such unhabitual postures of body parts have been reported to affect various perceptual processes (Eimer et al., 2001; Kennett et al., 2001; Eimer et al., 2004). Previous studies demonstrated that attention facilitated behavioral performance in individual sensory modalities. Furthermore, attentional increases were noted in the amplitude of early and middle latency components of event-related brain potentials (ERPs) elicited by somatosensory stimulation (Desmedt and Robertson, 1977; Garcia-Larrea et al., 1995; Eimer et al., 2002; van Velzen et al., 2002; Valeriani et al., 2003; Kida et al., 2004b,c; Forster and Eimer, 2005; Kida et al., 2006; Gherri and Eimer, 2008; Press et al., 2008; Adler et al., 2009; Keil et al., 2017; Novicic and Savic, 2023; Savic et al., 2023). Auditory spatial selective attention exerts two types of effects on N1 amplitude: the superimposition of another negativity (processing negativity, PN, or its negative difference between attended and unattended channels, Nd) and the enhancement of N1 itself (Hillyard et al., 1973; Naatanen et al., 1978; Naatanen, 2000), and visual spatial attention also exerts both of these effects on amplitudes in the N1-P2 latency range (Johannes et al., 1995; Hillyard et al., 1998; Hillyard and Anllo-Vento, 1998). Regarding somatosensory spatial attention, previous studies demonstrated that an increase in N140 amplitude by selective spatial attention was caused by the superimposition of PN (Michie et al., 1987; Garcia-Larrea et al., 1995; Kida et al., 2004b), whereas others reported an enhancement of the exogenous component (Josiassen et al., 1982). Somatosensory Nd has been used to extract attentional modulations under different conditions (Eimer and Driver, 2000; Eimer et al., 2001; Eimer and Forster, 2003a). The modality-non-specific, late ERP component, P300, has been associated with the amount of attentional resource (Wickens et al., 1983; Kramer et al., 1985; Kida et al., 2004a, 2012a; Reuter et al., 2013; Munoz et al., 2014; Reuter et al., 2014; Akaiwa et al., 2022) as well as subjective probability and stimulus uncertainty (Johnson, 1986; Kok, 2001; Polich, 2020). However, the mechanisms by which somatosensory ERPs are modulated when attention is divided between different hands with and without overt motor responses and those by which the attentional modulation of ERPs is affected by hand position and arm posture have not yet been elucidated.

Previous studies in the auditory modality demonstrated that the amplitude of N1 in the divided attention condition was intermediate between those elicited by the attended and unattended channels during focused attention (Hink et al., 1977, 1978; Parasuraman, 1978). Regarding the distribution of attention, a gradient was detected in visual (Mangun and Hillyard, 1988; Wijers et al., 1989; Heinze et al., 1994), auditory (Teder-Salejarvi and Hillyard, 1998; Teder-Salejarvi et al., 1999), and somatosensory ERPs (Heed and Roder, 2010). Some studies reported somatosensory-specific findings on attentional selectivity and gradients using ERPs (Eimer and Forster, 2003b; Forster and Eimer, 2004) and MEG (Kida et al., 2018). Moreover, psychophysical studies showed that visual (LaBerge, 1983; Downing and Pinker, 1985; Shulman et al., 1985, 1986), auditory (Mondor and Zatorre, 1995; Rorden and Driver, 2001), and somatosensory attention (Craig, 1985; Evans et al., 1992; Rinker and Craig, 1994; Lakatos and Shepard, 1997) had a gradient. In addition to the early component, the amplitude of P300 has been considered to reflect the amount of the attentional resource, and the correlation of single-trial P300 latency with reaction times has been associated with the allocation of resources (Kida et al., 2004a, 2012b). Therefore, different ERP components may provide useful information on the effects of divided attention within the somatosensory modality at different stages. In consideration of divided attention to different body parts, somatosensory attention to a stimulus is closely associated with attention to an action regarding target locations (Gherri and Eimer, 2010), i.e., the target body parts, in contrast to other sensory modalities. Therefore, further studies are needed to establish whether focused and divided attention exert the same effects on somatosensory processing in both a motor (overt) response task and mental (covert) task.

The position of the hands and posture of the arms have been reported to affect behavioral performance and cortical activation regarding attentional processing (Eimer et al., 2001; Kennett et al., 2001; Eimer et al., 2004; Gherri and Forster, 2012a). Previous studies demonstrated that somatosensory ERPs were markedly affected by hand position and arm posture, with the attentional effect being smaller for crossed forearms than for uncrossed forearms (Eimer et al., 2001; Gherri and Forster, 2012a). Another ERP study employed a cue-target attention task to show that posterior late directing attention positivity (LDAP) elicited during the cue-target interval and the attentional enhancement of somatosensory N140 amplitude increased when the hands were wide apart (Eimer et al., 2004). Neural modulations by manipulating hand position and arm posture may be associated with an interaction or incongruence between anatomical and external spaces where body parts receive somatosensory inputs. A psychophysical study indicated that the gradient of somatosensory attention depended on the physical space, but not the anatomical space (Lakatos and Shepard, 1997), whereas ERP studies showed that ERP modulations by crossing the forearms were caused by an incongruency between different spatial coordinates (Eimer et al., 2001; Gherri and Forster, 2012a). Physical space is a three-dimensional extent in the physical world whereas anatomical space or reference frame is the extent based on the body of the perceiver. That is, the difference between the two spatial codes is whether these are based on the physical (external) world or our body (internal world). More concretely, anatomical space codes the location of a somatosensory stimulus according to a somatotopic map where specific body locations are determined by the position of the stimulated cutaneous receptors and their cortical representation (Gherri and Forster, 2012a). Hence, anatomical codes are independent of the position of the body in physical or external space. We here use these two terms to describe what kinds of spatial reference frame attention is coordinated in. These terms have been used in a number of previous studies (Eimer et al., 2001, 2004; Gillmeister and Forster, 2012; Gherri and Forster, 2012a,b). Modality-non-specific late ERP P300 may be sensitive to changes in hand position and arm posture, which yield an interaction between different sensory coordinates. The amplitude of P300 has been implicated in the allocation of modality-non-specific attentional resources (Wickens et al., 1983; Kramer et al., 1985; Kok, 2001; Kida et al., 2004a, 2012a,b) as well as post-stimulus uncertainty (Sutton et al., 1965; Johnson, 1986; Polich, 2007). Therefore, manipulations of hand position and arm posture may affect post-stimulus uncertainty or resource allocation, resulting in changes in P300 and behavioral measures as well as their association.

A classical technique, the adaptive correlation filter (Woody, 1967), has been used to estimate single-trial P300 latency in association with behavioral reaction times. The correlation of single-trial P300 latency with reaction times has been successfully used to examine the coupling and decoupling of stimulus- and response-related processes under various task conditions; e.g., speed-*vs*-accuracy task instructions (Kutas et al., 1977; Pfefferbaum et al., 1983) and dual-task performance (Kida et al., 2012b). These studies reported diverse findings, which may be explained by the task-dependent coupling modes of stimulus- and response-related processes. Therefore, this technique may effectively detect changes in stimulus–response coupling caused by focused vs. divided attention or by manipulating hand position and arm posture.

In a series of three experiments, we examined the effects of focused versus divided attention on somatosensory ERPs, and also the impact of hand position and arm posture on the effects of focused attention. The aims of the present study were to clarify (1) whether somatosensory attention divided between the hands produced the same pattern of modulation of ERPs as reported in other modalities, (2) whether the patterns of the attentional modulation of ERPs were similar between mental (covert) and motor (overt) target detection tasks, (3) whether the effects of somatosensory selective attention were based on anatomical or physical spaces or their interaction, (4) whether the modality-non-specific late component (P300) was more sensitive to changes in hand position and arm posture than that of an earlier component (N140), and (5) the mechanisms by which these factors, including the type of attention, hand position, and arm posture, affect stimulus–response coupling assessed by the correlation of single-trial P300 latency with reaction times.

## Materials and methods

**Figure 1:**
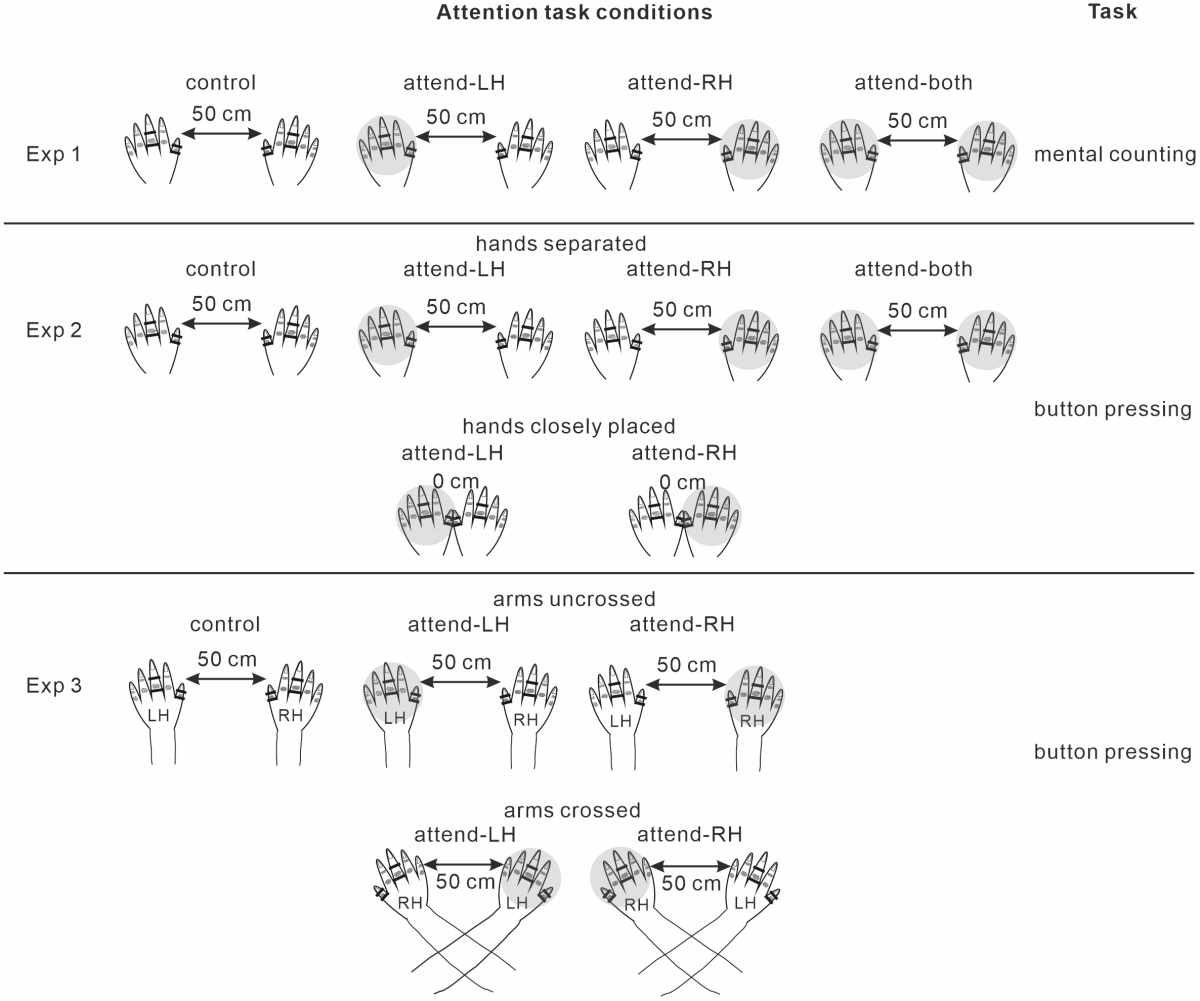
Attention task conditions in each experiment. Shaded is the to-be-attended hand. LH, left hand; RH, right hand.

The present study consisted of 3 experiments (Figure 1). Experiment 1 examined ERP modulations by focused and divided attention in a mental counting task. Experiment 2 investigated ERP modulations by focused and divided attention in a motor response task, and also ERP modulations by focused attention in different hand positions (closely-placed vs. separated hands). Experiments 1 and 2 were conducted in the uncrossed forearm position. Experiment 3 examined ERP modulations by focused attention in uncrossed and crossed forearm postures.

### Subjects

Ten right-handed healthy adults (1 female, 9 males), aged 22–30 years old, participated in experiment 1. Ten adults (1 female, 9 males), aged 23–30 years old, participated in experiment 2. Ten adults (2 female, 8 males), aged 23–30 years old, participated in experiment 3. In the present study, the inclusion criterion was an age of 20–40 years, while exclusion criteria were a history of neurological and psychiatric diseases, neurological surgery, and substance abuse. Some subjects participated in two or three experiments in a random order with at least a one-month interval between experiments. The present study was approved by the Ethics Committee, Graduate School of Comprehensive Human Sciences at the University of Tsukuba.

### Stimulation

Electrocutaneous stimuli (square wave, constant current pulse) of a 0.2-ms duration were presented to the left thumb (40%, standard) or middle finger (10%, deviant) and right thumb (40%, standard) or middle finger (10%, deviant) in a random order through ring electrodes attached to the first (anode) and second (cathode) interphalangeal spaces. The stimulus intensity was adjusted to approximately 2.5-fold the subject’s sensory threshold and was never reported as painful and uncomfortable. Interstimulus intervals varied randomly between 700 and 900 ms for 11 steps (mean 800 ms).

### Task condition

Subjects were seated comfortably in a chair in an electrically-shielded room, placed their hands with the palms down on a wooden board, and performed several attention conditions in each experiment. They were instructed to look at a crosshair 1.5 m in front of them and not to look at their hands during performance of the task. In all experiments, each condition consisted of 4 runs of approximately 200–300 stimuli, resulting in 1000 stimuli (400 left and 400 right standard stimuli and 100 left and 100 right deviant stimuli). The order of conditions was randomized among subjects. The interval between conditions was approximately 3 min, and that between runs was about 1 min. In all experiments, a control condition was performed, where subjects were instructed to relax and look at a crosshair 1.5 m in front of them and had no task. The control condition allowed us to assess whether ERPs were facilitated on the attended side or were suppressed on the unattended side (Alho et al., 1987; Desmedt and Tomberg, 1989, 1991; Garcia-Larrea et al., 1995; Kida et al., 2004b).

#### Experiment 1 (focused vs. divided attention in a mental counting task)

Each subject performed 4 different conditions including a control condition (Figure 1). In the attend-right condition, they silently counted the number of infrequent deviant stimuli (targets) presented to the right middle finger. In the attend-left condition, they silently counted the number of infrequent deviant stimuli presented to the left middle finger. The attend-right and attend-left conditions were regarded as the focused (or unilateral) attention conditions. In the divided attention condition, they counted the number of infrequent deviant stimuli presented to the right and left hands. The thumbs of the left and right hands were located separately at a distance of 50 cm. Subjects were asked to report the number after the termination of each run. The number of target stimuli slightly differed (difference of 0–6) among the runs to prevent subjects from assuming the number without counting; however, each condition consisted of the same number of target (deviant) stimuli.

#### Experiment 2 (focused vs. divided attention and effect of hand position in a motor response task)

This experiment was the same as experiment 1, except for the type of target detection and manipulation of hand position. Each subject performed 5 different conditions where the direction of attention and hand position varied, plus a control condition (a total of 6 task conditions). Subjects were instructed to press a button with the index finger as fast as possible when they detected infrequent deviant stimuli presented to the left or right middle finger, respectively, in two focused attention (attend-left or right) conditions with the same hand position as that in experiment 1 (i.e., with a 50-cm inter-hand distance). In the divided attention condition, they responded to infrequent deviant stimuli to the right and left hands by pressing a button. In the other 2 conditions, the thumbs were located close to each other such that they were almost in contact, and subjects performed the same task as focused attention conditions. Button pressing was conducted with compatible mapping within the somatosensory modality, irrespective of hand position (i.e., motor response with the right hand to the right-hand stimulus, and motor response with the left hand to the left-hand stimulus, irrespective of hand position).

#### Experiment 3 (effects of crossing forearms)

Each subject performed 4 attention conditions plus a control condition (a total of 5 conditions). Attend-right and attend-left conditions were performed with the hands located separately at a distance of approximately 50 cm, as in experiments 1 and 2 (uncrossed-forearms condition). In the other 2 conditions, attend-right and attend-left conditions were performed with the forearms crossed (crossed-forearms condition). Subjects were instructed to detect infrequent stimuli on the designated side by button pressing, which was conducted with compatible mapping within the somatosensory modality, irrespective of forearm postures.

### Recordings and analysis

Electroencephalograms (0.5–100 Hz) were recorded at a sampling rate of 500 Hz with Ag/AgCl electrodes from 5 locations on the scalp: Fz, Cz, Pz, C3, and C4 (SYNAFIT, Nihon Denki San-ei Corp., Japan). All the electrodes were referenced to the average of earlobes. Impedance was carefully balanced and maintained below 5 kohm. Electrooculograms (EOG) were recorded bipolarly from the right outer canthus and suborbital region to monitor eye movements or blinks. The analysis time was 600 ms, including a 50-ms prestimulus baseline. Trials exceeding ±80 μV (EOG and EEG amplitudes) were automatically excluded from averaging, and trials with eye blinks and eye movements were excluded manually. Trials with omission and commission errors were also excluded from further analyzes. Grand-averaged waveforms were filtered using a low-pass Butterworth filter with a cut-off frequency of 40 Hz.

#### Count accuracy

Count accuracy (CA) in experiment 1 was computed in each run using the following equation: CA = 100–100*(abs(correct count – subject’s count)/correct count), and then averaged across 4 runs in each condition. A one-way repeated measures analysis of variance (ANOVA) was performed on CA with attention (3 levels; Attend-right, attend-left, and both) as a factor.

#### Reaction times and response accuracy

The reaction time (RT) was measured between 100 and 550 ms after the onset of target stimuli in the button-pressing task in experiments 2 and 3. Response accuracy (RA) was computed using the following equation: RA = 100*((target number-missed target number)/target number). A two-way ANOVA was performed separately on RT and RA with attention (3 levels; focused/hands-separated, focused/closely-spaced hands, and divided) and the stimulus hand (2 levels; left and right) as factors in experiment 2. In experiment 3, a two-way ANOVA was performed separately on RT and RA with forearm posture (crossed and uncrossed) and the stimulus hand (left and right) to compare the effects of forearm posture on behavioral measures.

#### Statistical analysis of ERPs

N140 was observed for both standard and deviant stimuli, in contrast to only P300 for target stimuli. The peak amplitude and latency of N140 at Fz and the contralateral central site (the average between C3 for right-hand stimulation and C4 for left-hand stimulation) and P300 at Pz were measured within time windows of 110–180 and 250–550 ms, respectively. Time windows were selected based on previous studies that investigated these ERP components (Kida et al., 2004a,b, 2012a,b). N140 is considered to be generated in frontal areas, such as the anterior cingulate cortex (Tanaka et al., 2008), supplementary motor area (Allison et al., 1992), and second somatosensory cortex (Tarkka et al., 1996; Valeriani et al., 2000), and, thus, is recorded maximally at the frontal and central midline electrodes. Furthermore, in consideration of the involvement of the post-central region, some studies examined N140 at the contralateral central electrode (Eimer and Driver, 2000; Kennett et al., 2001; Eimer et al., 2003b; Forster and Eimer, 2004; Gherri et al., 2023). Some source modeling studies reported the partial generation of N140 in the parietal cortex, such as SI (Valeriani et al., 2001). Based on these findings and the distribution of data in the present study, we focused on data obtained from the Fz and contralateral central electrodes. P300 generally showed a broad distribution, with the maximal amplitude being obtained at the parietal electrode in all modalities. In our experience, somatosensory P300 showed the maximal amplitude at the central (Cz) and parietal (Pz) electrodes (Kida et al., 2003a,b,c, 2004a, 2012b). Therefore, we focused on P300 data obtained from Pz in the present study. In each of experiments 1 and 2, for the amplitudes of N140, a two-way ANOVA was performed with condition (control, focused, unattended, and divided) and stimulus type (standard vs. deviant) as factors. In experiment 2, we also performed a three-way ANOVA of N140 amplitude with condition (attended vs. unattended), hand position (closely-placed vs. separated), and stimulus type (standard vs. deviant). In experiment 3, we performed a three-way ANOVA of N140 amplitude with condition (attended vs. unattended), forearm posture (crossed vs. uncrossed), and stimulus type (standard vs. deviant). P300 was observed for attended deviant (target) stimuli in the focused attention and divided attention conditions, whereas unattended deviant stimuli in the focused attention condition, identical deviant stimuli in the control condition, and standard stimuli did not elicit P300. Therefore, a paired *t*-test was performed to compare the amplitude or latency of P300 between focused attention and divided attention conditions, between hand positions (closely-placed vs. separated), and between forearm postures (crossed vs. uncrossed). In ANOVA, if the assumption of sphericity was violated in Mauchly’s test, the degree of freedom was corrected using the Greenhouse–Geisser correction coefficient epsilon, and the value of p was then recalculated. The significance level was set at *p* < 0.05. A multiple comparison test with Šidák correction was used for the post-hoc analysis. Therefore, the reported value of p in the multiple comparison test was based on an adjusted value computed backward; i.e., the adjusted value of p, *p(adjusted)*, was computed using the following equation: *p(adjusted) = 1-(1-p(unadjusted)^c)*, where *c* is the comparison number. The Šidák correction was applied to multiple comparisons, including behavioral and ERP data. Partial-eta squared (η_p_^2^) was computed as an effect size measure in ANOVA. Cohen’s *d* was also reported as an effect size measure in the paired *t*-test.

#### Analysis of single-trial P300 latency and RT

We used an adaptive correlation filter method to estimate single-trial P300 latency (Woody, 1967; Kutas et al., 1977; Kida et al., 2012b). Data measured at Pz in response to target stimuli were used in this analysis. We followed our previous analysis procedure for this technique (Kida et al., 2012b). We selected only the trials showing a cross-correlation coefficient, R > 0.80, in the final template, i.e., we regarded these trials as good estimates. The correlation coefficient between single-trial P300 latency and RT was examined in each condition. Cohen’s *q* was computed as an effect size measure for the significance of differences between two correlations.

## Results

### Experiment 1 (focused vs. divided attention in a mental counting task)

N140 amplitude was increased more by focused attention than by the other conditions examined. Furthermore, N140 amplitude in the divided attention condition was intermediate between the focused and unattended conditions. P300 amplitude was lower in the divided attention condition than in the focused attention condition. Detailed information is provided below.

#### Behavioral data

CA was slightly lower when attention was divided between hands than when it was focused on one hand (Table 1) (*F* (2, 18) = 1.7, *η*_p_^2^ = 0.16).

**Table 1:** Means (±SE) of count accuracy (CA) in the silent counting task in experiment 1.

	LH target	RH target	Divided attention
CA (%)	98.6 (0.8)	98.7 (0.6)	97.3 (1.1)

LH, left hand; RH, right hand.

#### ERPs

**Figure 2:**
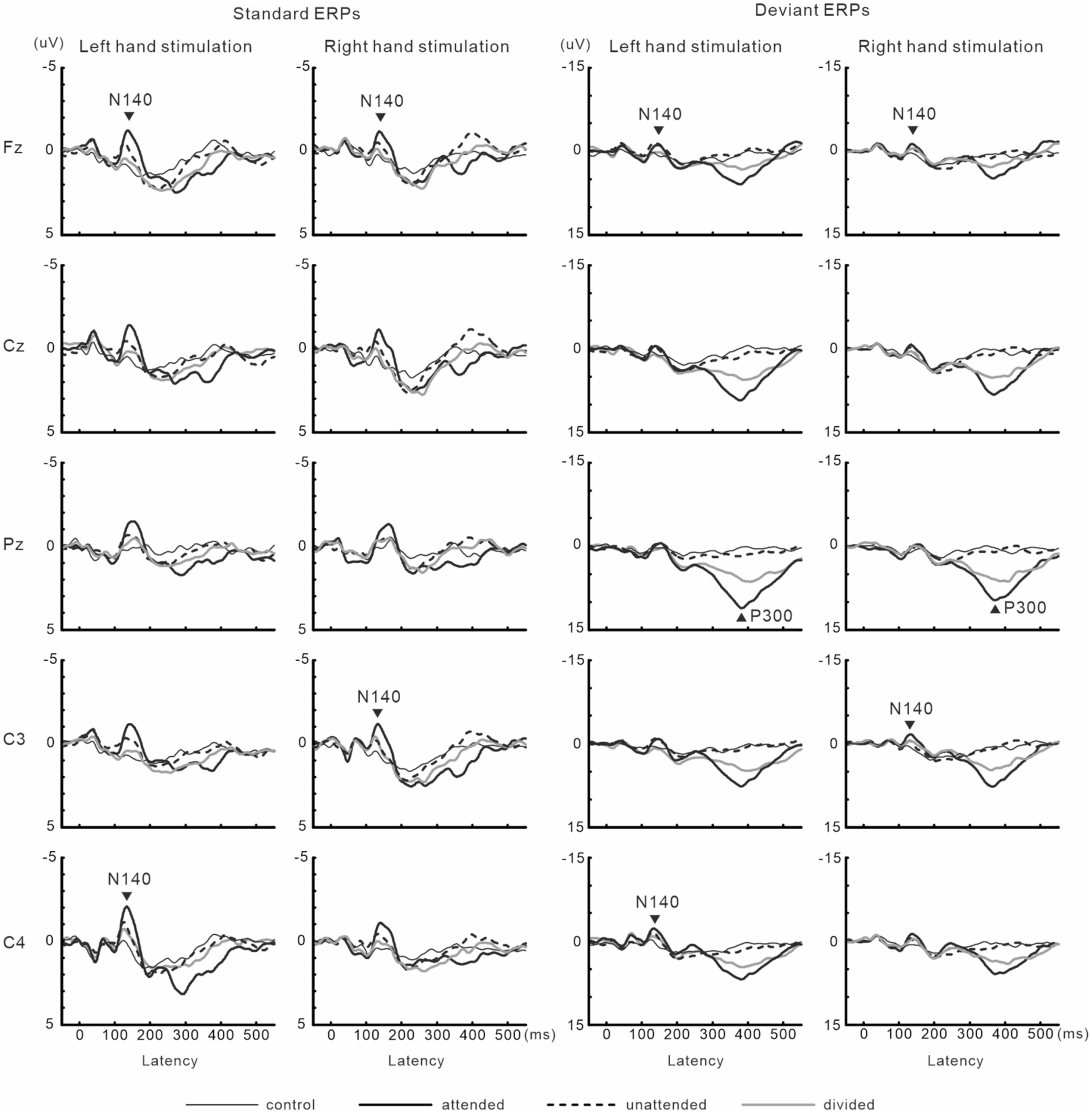
Grand-averaged waveforms of ERPs elicited by standard and target stimuli in experiment 1.

**Figure 3:**
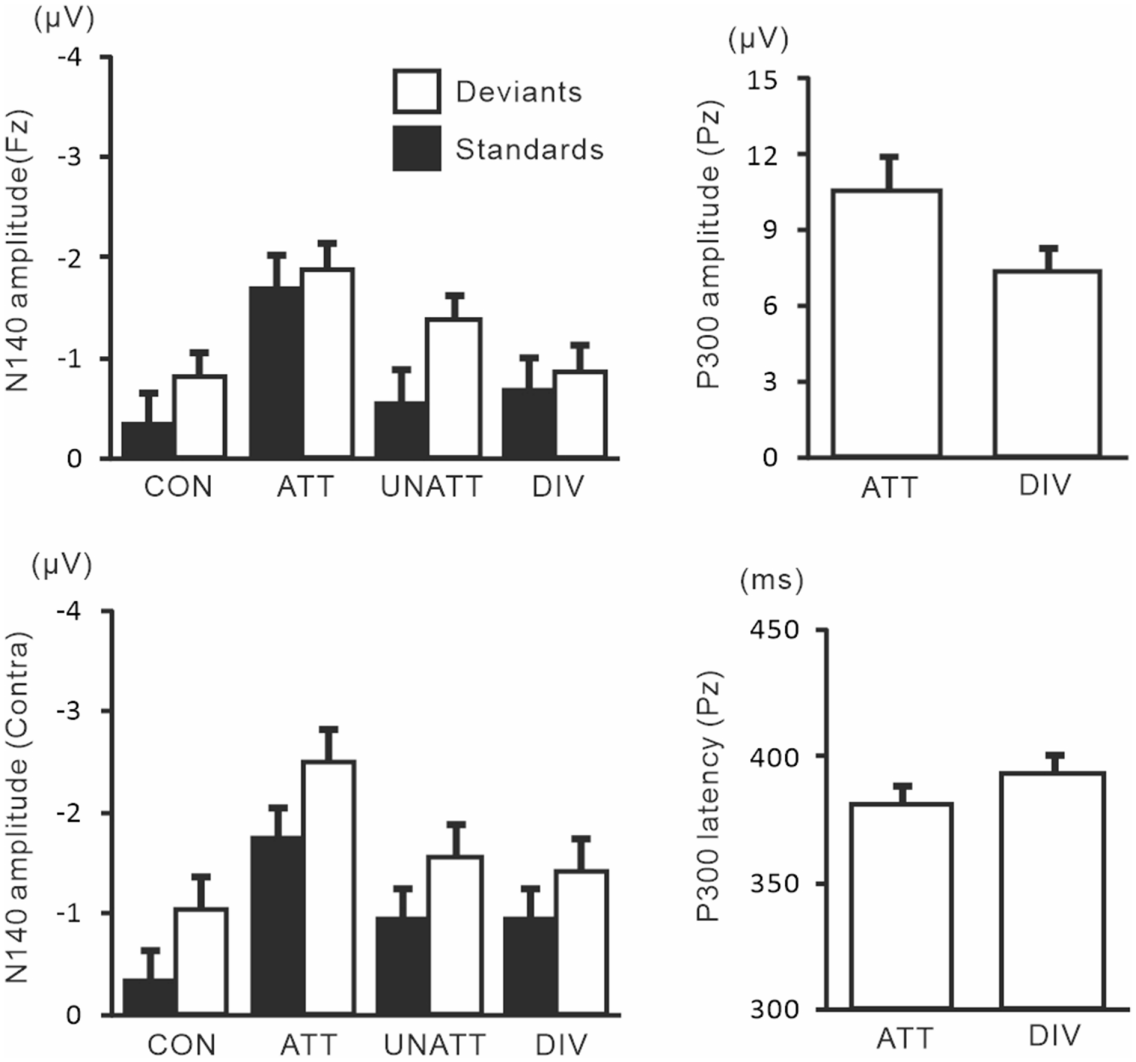
Mean values of ERP amplitudes and latencies across subjects in experiment 1. ERP modulations are shown for comparison between unilaterally focused vs. divided attention conditions in a mental counting task. The amplitudes and latencies averaged across left-and right-hand stimuli are shown here because there was no main effect of the stimulus hand and no interaction, including the stimulus hand factor. CON, control condition; ATT, attended stimuli; UNATT, unattended stimuli; DIV, divided attention condition; Contra, contralateral central electrode to stimulation.

N140 and P300 components were detected in all subjects (Figure 2). In the two-way ANOVA of N140 amplitude with attention (control, attended, unattended, and divided) and stimulus type (standard vs. deviant), there were significant main effects of the attention condition (*F* (3, 27) = 7.5, *p* < 0.001, *η*_p_^2^ = 0.69 at Fz; *F* (3, 27) = 9.7, *p* < 0.001, *η*_p_^2^ = 0.52 at the contralateral central site) and stimulus type (*F* (1, 9) = 20.3, *p* < 0.001, *η*_p_^2^ = 0.69 at Fz; *F* (1, 9) = 13.6, *p* < 0.005, *η*_p_^2^ = 0.60 at the contralateral central site). N140 amplitude was higher in the focused attention condition than in the control (*p* < 0.001), unattended (*p* < 0.05), and divided attention conditions (*p* < 0.05) (Figure 3). N140 amplitude in the divided attention condition was intermediate between the focused attention and unattended conditions. The analysis of the effect of stimulus type showed that deviant stimuli elicited larger N140 than standard stimuli. The interaction between the stimulus type and attention condition was not significant for N140 amplitude. P300 amplitude was significantly lower in the divided attention condition than in the focused attention condition (*t* = 4.1, *p* < 0.005), whereas its latency was not significantly changed (*t* = 1.1) (Figure 3).

### Experiment 2 (focused vs. divided attention and closely-placed vs. separated hands in a motor response task)

**Figure 4:**
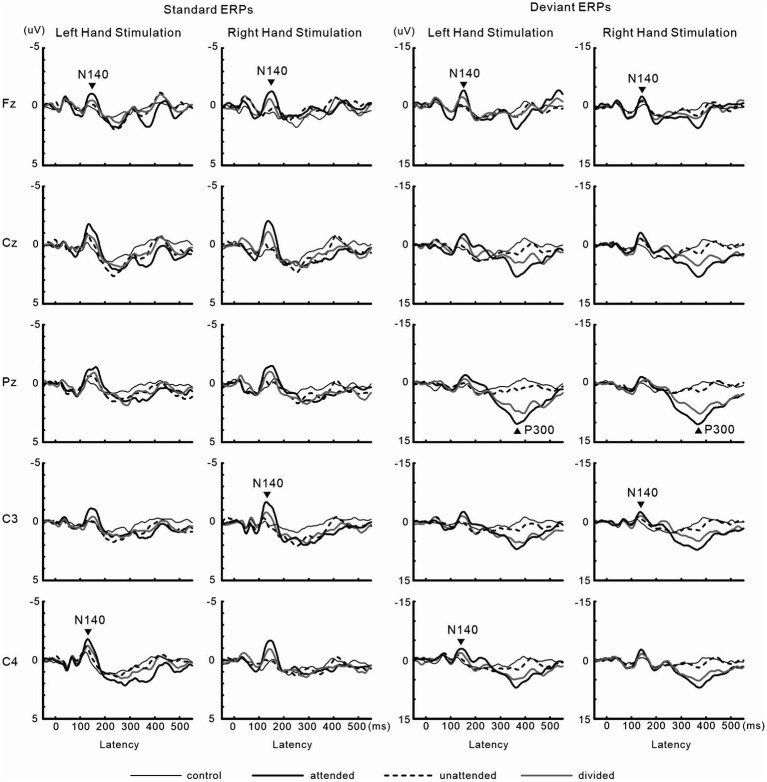
Grand-averaged waveforms of ERPs elicited by standard and target stimuli in experiment 2. Data in the hands closely-placed condition are not displayed here, but are shown in Figure 6.

The effects of focused and divided attention on ERP amplitude were similar to those in experiment 1 (Figure 4). P300 latency was longer in the divided attention condition than in the focused attention condition. Hand position did not affect N140, but changed P300 amplitude and latency. Detailed information is provided below.

#### Behavioral data

The two-way ANOVA of RT showed a significant main effect of attention (*F* (2, 9) = 4.7, *p* < 0.05, *η*_p_^2^ = 0.34), such that RT was significantly longer when attention was divided between the hands than when it was focused on one hand (*p* < 0.05) (Table 2). The two-way ANOVA of RA showed a significant main effect of attention (*F* (2, 18) = 7.2, *p* < 0.01, *η*_p_^2^ = 0.45), such that RA was lower when attention was divided between the hands than when it was focused on one hand (*p* < 0.05) or when the hands were closely placed than when separated (*p* < 0.005) (Table 2). There was no significant interaction for RT or RA.

**Table 2:** Means (±SE) of the reaction time (RT) and response accuracy (RA) in a button-pressing task in experiment 2.

	Focused (unilateral) attention				Divided attention	
	Hands separated		Hands closely placed			
	LH target	RH target	LH target	RH target	LH target	RH target
RT (ms)	376.3 (14.2)	371.7 (14.8)	374.5 (14.5)	369.1 (15.6)	394.7 (13.8)*	393.3 (11.4)*
RA (%)	95.7 (1.2)	96.9 (0.9)	90.7 (1.4)*	92.2 (1.9)*	90.3 (2.6)*	87.9 (3.3)*

*p < 0.05, vs. Focused attention/Hands separated.

#### ERPs

**Figure 5:**
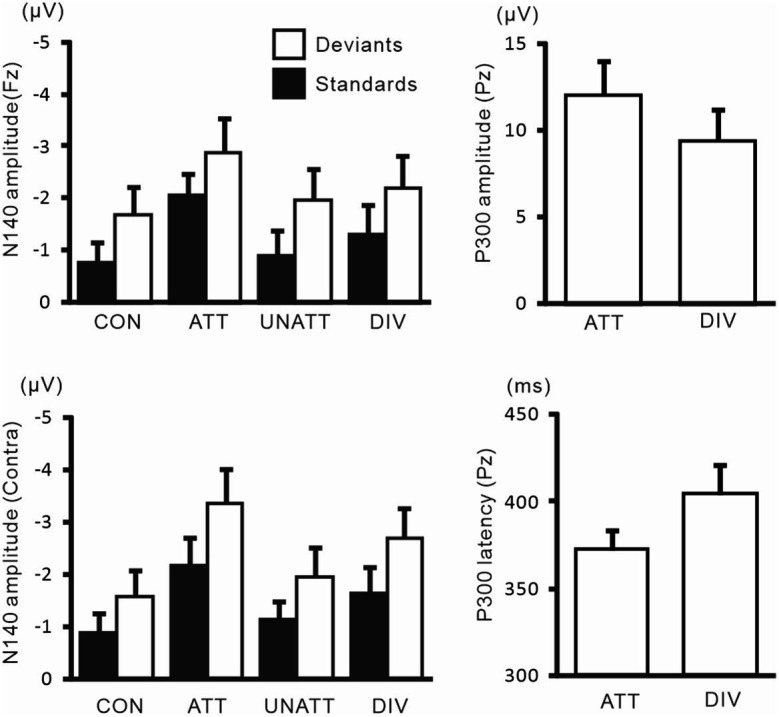
Mean values of ERP amplitudes and latencies across subjects in experiment 2. ERP modulations are shown for comparison between unilaterally focused vs. divided attention conditions in a motor response task. Amplitudes and latencies averaged across left-and right-hand stimuli are shown.

In the two-way ANOVA of N140 amplitude with attention (control, attended, unattended, and divided) and stimulus type (standard vs. deviant), there were significant main effects of the attention condition (*F* (3, 27) = 20.9, *p* < 0.001, *η*_p_^2^ = 0.69 at Fz; *F* (3, 27) = 11.4, *p* < 0.001, *η*_p_^2^ = 0.56 at the contralateral central site) and stimulus type (*F* (1, 9) =8.1, *p* < 0.01, *η*_p_^2^ = 0.47 at Fz; *F* (1, 9) = 14.1, *p* < 0.05, *η*_p_^2^ = 0.61 at the contralateral central site). N140 amplitude was higher in the focused attention condition than in the control (*p* < 0.001 at Fz and *p* < 0.05 at the contralateral central site), unattended (*p* < 0.05 at Fz and p < 0.005 at the contralateral central site), and divided attention conditions (*p* < 0.05) (Figure 5). N140 amplitude in the divided attention condition was intermediate between the focused and unattended conditions. The analysis of the stimulus type effect showed that deviant stimuli elicited larger N140 than standard stimuli. Regarding N140 amplitude, there was a significant interaction between the stimulus type and attention condition (*F* (3, 27) = 3.4, *p* < 0.05, *η*_p_^2^ = 0.27 at the contralateral central site), such that the amplitude was higher for focused attention and divided attention standard stimuli than for unattended standard stimuli, whereas it was higher for focused attention deviant (target) stimuli than deviant stimuli in the control condition and unattended deviant stimuli. P300 amplitude was significantly lower (*t* = 3.7, *p* < 0.005) and latency was longer (*t* = 3.9, *p* < 0.005) in the divided attention condition than in the focused attention condition.

**Figure 6:**
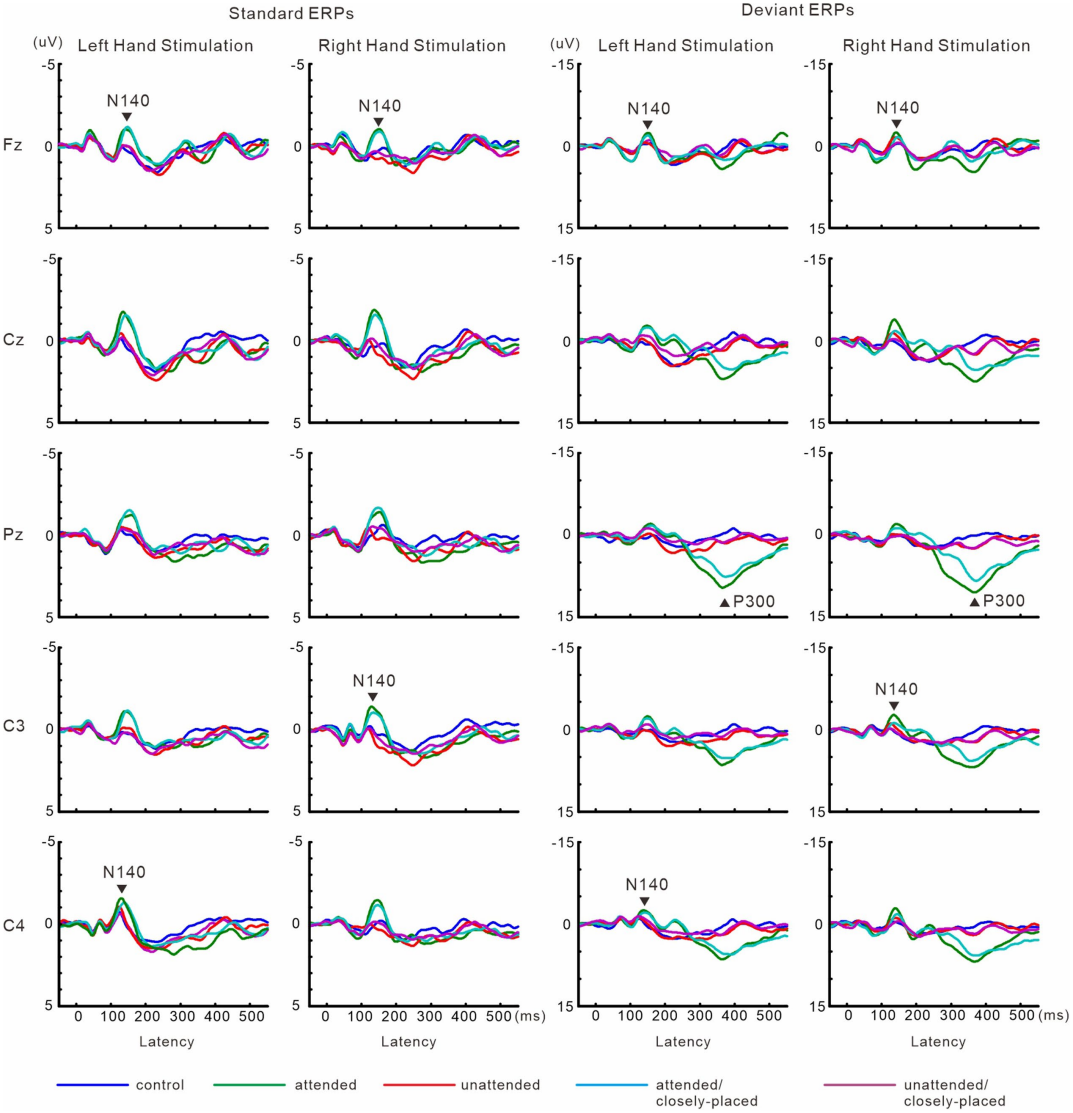
Grand-averaged waveforms of ERPs elicited by standard and target stimuli in experiment 2. Data for attended and unattended stimuli in the hands-separated condition, which are identical to that for attended and unattended stimuli in Figure 4, are shown here for comparison with the hands closely-placed condition.

**Figure 7:**
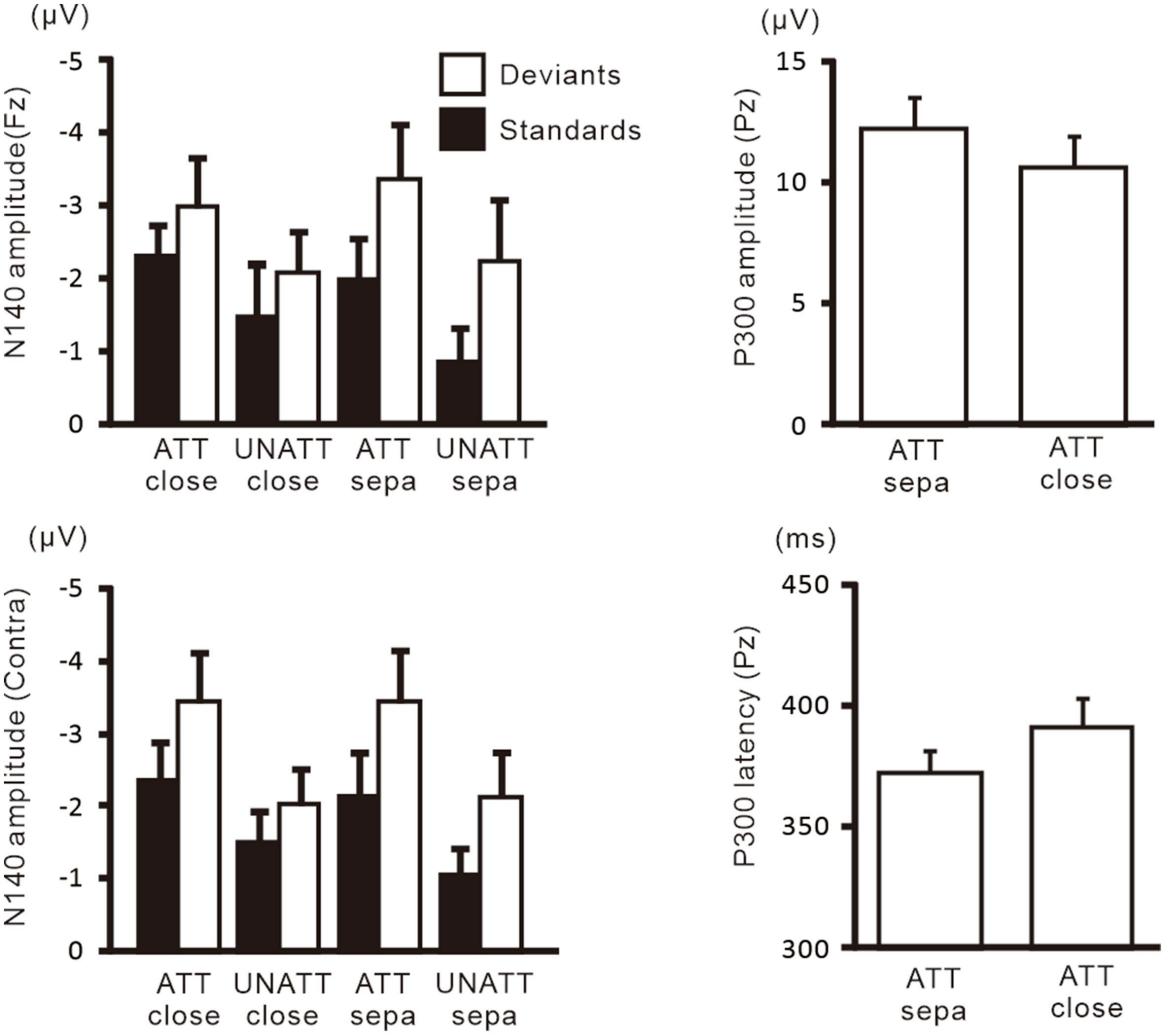
Mean values of ERP amplitudes and latencies across subjects in experiment 2. Data for attended and unattended stimuli in the hands-separated condition, which are identical to that for attended and unattended stimuli in Figure 5, are shown here for comparison with the hands closely-placed condition. Amplitudes and latencies averaged across left-and right-hand stimuli are shown. Close, hands closely-placed; sepa, hands-separated.

Figure 6 shows grand-averaged ERP waveforms in the hands closely-placed and separated conditions. We performed a 3-way ANOVA of N140 amplitude with attention (attended and unattended), hand position (closely-placed vs. separated), and the stimulus type (standard and deviant) to examine the impact of hand position on somatosensory processing and attention effect. There were significant main effects of the attention condition (*F* (1, 9) = 31.8, *p* < 0.001, *η*_p_^2^ = 0.78 at Fz; *F* (1, 9) = 15.3, *p* < 0.005, *η*_p_^2^ = 0.63 at the contralateral central site) and stimulus type (*F* (1, 9) = 11.5, *p* < 0.01, *η*_p_^2^ = 0.56 at Fz; *F* (1, 9) = 20.2, *p* < 0.005, *η*_p_^2^ = 0.69 at the contralateral central site) (Figure 7). There was also a significant main effect of hand position at the contralateral central site (*F* (1, 9) = 8.2, *p* < 0.05, *η*_p_^2^ = 0.47), but no significant interactions, including hand position. P300 amplitude was significantly lower (*t* = 2.3, *p* < 0.05) and latency was significantly longer (*t* = 2.4, *p* < 0.05) when the hands were closely placed than when they were wide apart.

### Experiment 3 (crossed vs. uncrossed forearms)

**Figure 8:**
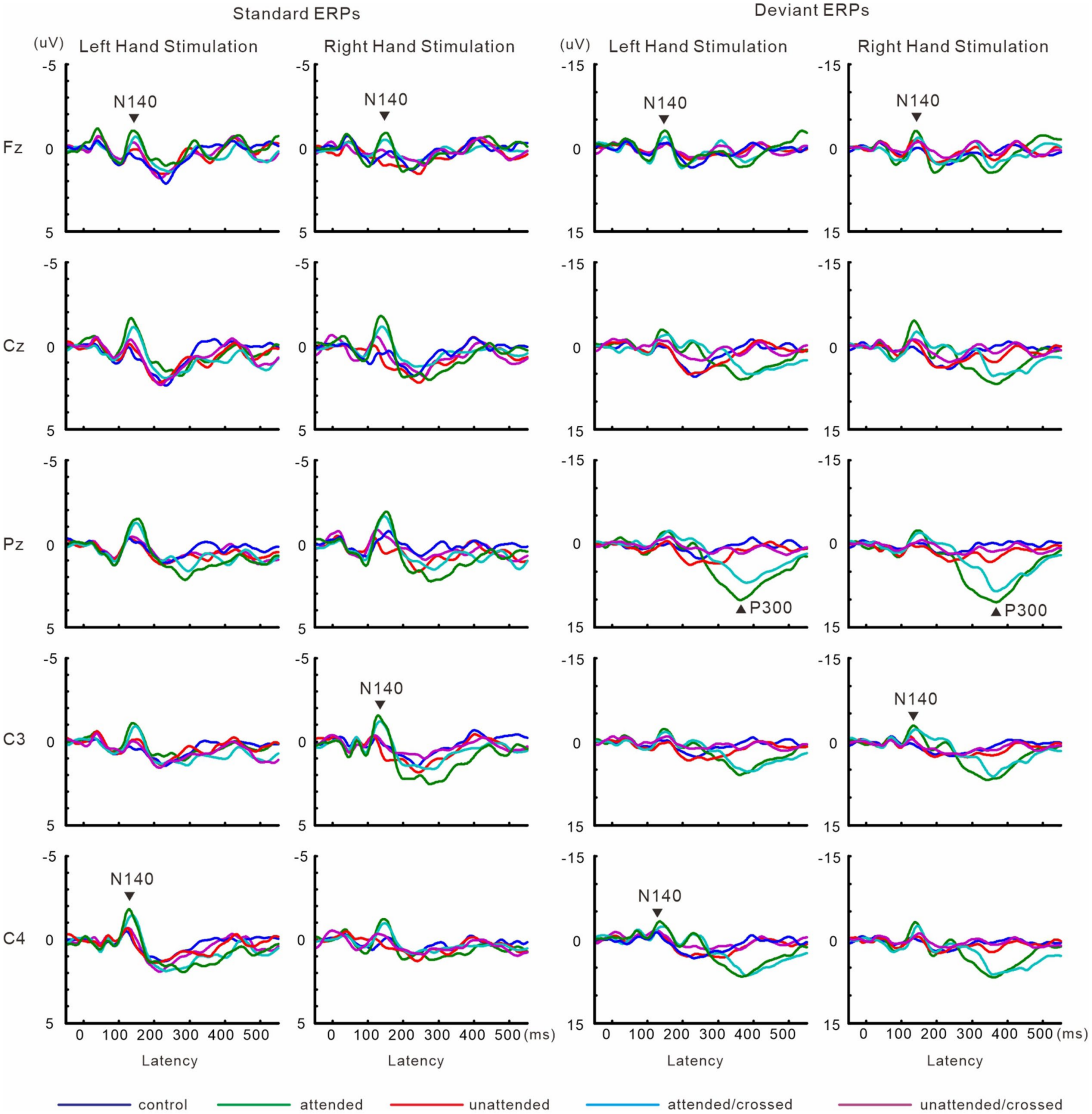
Grand-averaged waveforms of ERPs elicited by standard and target stimuli in experiment 3.

Figure 8 shows grand-averaged ERP waveforms in experiment 3. The effect size of focused attention on N140 amplitude was changed by crossing the forearms. Forearm posture affected P300 amplitude and latency. Detailed information is provided below.

#### Behavioral data

Regarding RT, there was a main effect of forearm posture (*F* (1, 9) = 7.3, *p* < 0.05, *η*_p_^2^ = 0.34), with RT being longer when the forearms were crossed than when they were uncrossed independent of the stimulus hand (Table 3). Concerning RA, there was a main effect of forearm posture (*F* (1, 9) = 18.2, *p* < 0.005, *η*_p_^2^ = 0.67), with RA being lower when the forearms were crossed than when they were uncrossed independent of the stimulus hand. No interaction was found for RA.

**Table 3:** Means (±SE) of RT and RA in the button-pressing task in experiment 3.

	Forearms uncrossed		Forearms crossed	
	LH target	RH target	LH target	RH target
RT (ms)	369.7 (14.6)	363.5 (15.4)	377.1 (15.1)*	376.9 (16.3)*
RA (%)	95.6 (1.2)	96.1 (1.3)	90.2 (2.0)*	90.6 (2.2)*

*p < 0.05, vs. Forearms uncrossed.

#### ERPs

**Figure 9:**
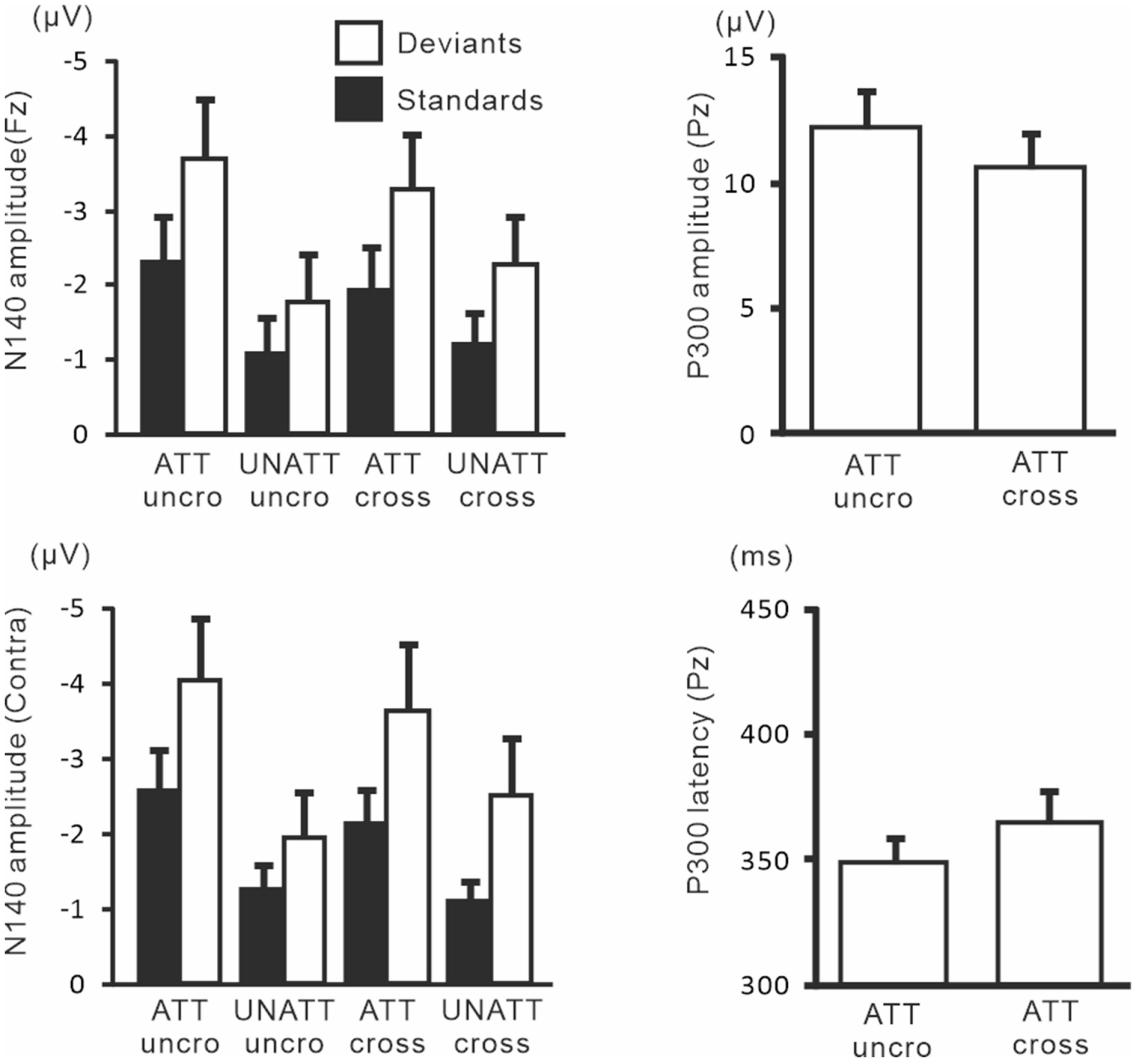
Mean values of ERP amplitudes and latencies across subjects in experiment 3. ERP modulations are shown to compare crossed- vs. uncrossed-forearms conditions. Amplitudes and latencies averaged between left-and right-hand stimuli are shown. Cross, crossed forearms; uncro, uncrossed forearms.

Figure 9 shows the amplitudes and latencies of ERPs in Experiment 3. In the 3-way ANOVA of N140 amplitude, there was a significant main effect of the attention condition (*F* (1, 9) = 21.9, *p* < 0.05, *η*_p_^2^ = 0.71 at Fz; *F* (1, 9) = 18.5, *p* < 0.01, *η*_p_^2^ = 0.67 at the contralateral central site), with amplitude being higher when attention was directed to one hand than with unattended stimuli. The stimulus type effect was also significant (*F* (1, 9) = 11.8, *p* < 0.01, *η*_p_^2^ = 0.57 at Fz; *F* (1, 9) = 12.8, *p* < 0.01, *η*_p_^2^ = 0.59 at the contralateral central site). The interaction including forearm posture was not significant on N140 amplitude at both the Fz and contralateral central sites; however, low-level multivariate ANOVAs showed that the effect size of attention was the highest for standard stimuli with uncrossed forearms (*F* (1, 9) = 20.4, *p* < 0.005; *η*_p_^2^ = 0.69), intermediate for standard stimuli with crossed forearms (*F* (1, 9) = 10.4, *p* < 0.01; *η*_p_^2^ = 0.55), and the lowest (but generally a moderate effect) for deviant stimuli with uncrossed (*F* (1, 9) = 7.5, *p* < 0.05; *η*_p_^2^ = 0.46) and crossed forearms (*F* (1, 9) = 6.9, *p* < 0.05; *η*_p_^2^ = 0.44). P300 amplitude was lower (*t* = 2.6, *p* < 0.05) and its latency was longer (*t* = 3.5, *p* < 0.01) when the forearms were crossed than when they were uncrossed.

### Single-trial P300 latency and RT

In the analysis of single-trial P300 latency using the ACF technique, 38.6 and 39.9% of all trials were identified as good estimates in experiments 2 and 3, respectively. In experiment 2, the two-way ANOVA (3 attention conditions [unilaterally-focused/hands-separated, unilateral focused/hands closely-placed, and divided] and 2 target sides [left-and right-hand targets]) of single-trial P300 latency showed a main effect of the attention condition (*F* (2, 18) = 4.4, *p* < 0.05; *η*_p_^2^ = 0.33), similar to the analysis of the averaged waveform, with latency being longer with hands closely-placed than separated (Table 4). Divided attention also resulted in slightly longer latency (Table 4) than unilaterally-focused attention. This was the same pattern as that observed in the results on averaged waveforms. The single-trial analysis of RT and P300 latency showed that RT preceded P300 latency in 46.0, 36.9, and 36.6% of good trials in the focused/hands-separated, focused/hands closely-placed, and divided attention conditions, respectively, for left-hand targets, and in 54.6, 43.1, and 39.2%, respectively, for right-hand targets. Therefore, right-hand targets had more trials with RT preceding P300 latency than left-hand targets in all conditions tested, and also hands closely-placed and divided attention decreased the preceding ratio of RT (or the delayed ratio of P300), resulting in a decrease in the difference between left-and right-hand targets. The variabilities (SD) of single-trial P300 latency and RT were not significant in ANOVA with the attention condition and target side. The correlation of single-trial P300 latency with RT was moderate in the unilaterally-focused attention/hands-separated condition (correlation coefficient *R* = 0.52) and low in the unilaterally-focused attention/hands closely-placed condition (*R* = 0.38) and divided attention condition (*R* = 0.38), all of which showed correlations (Table 4). The former showed a stronger correlation than the latter two.

**Table 4:** Mean (±SE) and SD of single-trial P300 latency, the SD of RT, the ratio of trials with RT preceding single-trial P300 latency, and the correlation of single-trial P300 latency with RT in experiment 2.

	Focused (unilateral) attention				Divided attention	
	Hands separated		Hands closely placed			
	LH target	RH target	LH target	RH target	LH target	RH target
Mean (ms) of single-trial P300 latency	340.0 (3.3)	338.3 (4.1)	349.4 (4.6)**	357.9 (6.9)**	344.6 (6.2)	342.6 (7.4)
Mean of SD of single-trial P300 latency	59.2 (5.8)	61.9 (5.3)	59.6 (5.8)	65.8 (9.9)	63.7 (3.2)	63.9 (4.0)
Mean of SD of single-trial RT	58.9 (4.7)	58.7 (4.3)	55.7 (4.2)	59.4 (4.0)	62.1 (2.6)	61.8 (3.2)
Ratio (%) of trials with RT preceding P300 latency	46.0	54.6	36.9	43.1	36.6	39.2
Correlation of single-trial P300 latency with RT	0.40*	0.52*	0.31*	0.44*	0.34*	0.44*
Correlation of single-trial P300 latency with RT (based on data concatenated across LH and RH)	0.47*		0.38*		0.38*	

*Correlation, *p* < 0.05, vs. zero; **p < 0.05, vs. Focused attention/Hands separated.

Data shown here were calculated after excluding bad trials (using the ACF technique), trials with missed targets, and trials with artifacts.

In experiment 3, the two-way ANOVA of (2 forearm positions and 2 target sides) single-trial P300 latency showed a main effect of forearm posture (*F* (1, 9) = 6.1, *p* < 0.05; *η*_p_^2^ = 0.41), with latency being longer when the forearms were crossed than when they were uncrossed irrespective of the target stimulus hand (Table 5). Therefore, the same pattern of results was observed as averaged waveforms. The single-trial analysis of RT and P300 latency showed that RT preceded P300 latency in 42.5 and 50.7% of good trials in the uncrossed-and crossed-forearms conditions, respectively, for left-hand targets, and in 59.3 and 51.3%, respectively, for right-hand targets (Table 5). Therefore, right-hand targets had more trials with RT preceding P300 latency in both forearm positions, and crossing the forearms decreased the RT-P300 latency difference between left-and right-hand targets. The variabilities (SD) of single-trial P300 latency and RT were not significant in ANOVA with forearm position and the target stimulus hand. A correlation of single-trial P300 latency with RT was observed in the uncrossed-forearms condition (*R* = 0.37, significant), but not in the crossed-forearms condition (*R* = 0.23). Furthermore, a significant difference was observed in the correlation between the two conditions (*z* = 1.98) with a small effect (*q* = 0.15). When it was examined separately for each target side, the correlation was significantly higher in the uncrossed-forearms condition for right-hand targets (*R* = 0.45) than in the crossed-forearms condition (*R* = 0.23) (*z* = 2.29, *p* < 0.05) with a small effect (*q* = 0.25), whereas left-hand targets showed no significant difference between the crossed- and uncrossed-forearms conditions.

**Table 5:** Mean (±SE) and SD of single-trial P300 latency, the SD of RT, the ratio of trials with RT preceding single-trial P300 latency, and the correlation of single-trial P300 latency with RT in experiment 3.

	Forearms uncrossed		Forearms crossed	
	LH target	RH target	LH target	RH target
Mean (ms) of single-trial P300 latency	335.3 (4.9)	335.8 (5.6)	358.6 (7.8)**	363.8 (10.9)**
Mean of SD of single-trial P300 latency	57.8 (7.1)	61.3 (5.1)	61.8 (5.8)	63.4 (6.7)
Mean of SD of single-trial RT	56.7 (4.4)	55.8 (3.3)	59.9 (3.7)	57.2 (4.0)
Ratio (%) of trials with RT preceding P300 latency	52.5	55.3	50.7	51.3
Correlation of single-trial P300 latency with RT	0.32*	0.45*	0.24	0.23**
Correlation of single-trial P300 latency with RT (based on data concatenated across LH and RH)	0.37*		0.23**	

*Correlation, *p* < 0.05, vs. zero; **p < 0.05, vs. Forearms uncrossed.

Data shown here were calculated after excluding bad trials (using the ACF technique), trials with missed targets, and trials with artifacts.

Another result on the correlation of single-trial P300 latency with RT was observed when left-and right-hand target stimuli were separately analyzed. The single-trial P300 latency-RT correlation in most of the conditions examined in experiments 2 and 3 was slightly stronger for right-hand target stimuli than for left-hand target stimuli (the only exception was the crossed-forearms condition), whereas no significant difference was observed when it was tested separately in each condition (Tables 4, 5). To increase the statistical power and examine correlation patterns general to all attention conditions, we concatenated latency and RT data from all attention conditions for each target side and then compared correlations between different target sides. The analysis revealed correlations for both target sides (*R* = 0.31 for left-hand target, *R* = 0.41 for right-hand target) and a significant difference in the correlation between left-and right-hand target stimuli (*z* = 2.28, *p* < 0.05) with a small effect (*q* = 0.12). Collectively, correlation patterns showed that right-hand attended targets (and responses) produced a stronger P300 latency-RT correlation with a small effect than left-hand targets, which was decreased by crossing the forearms.

## Discussion

### Replication of the attentional modulation of N140

The present study showed that the amplitude of N140 was modulated by directing attention to the unilateral hand, and was higher for attended stimuli than for unattended stimuli and identical stimuli in the control condition. Therefore, we successfully replicated previous findings on the effects of somatosensory attention on the amplitude of N140 (Desmedt and Robertson, 1977; Desmedt et al., 1984; Michie et al., 1987; Desmedt and Tomberg, 1989; Garcia-Larrea et al., 1995; Eimer and Forster, 2003a; Kida et al., 2004b, 2012a,b).

### Effects of divided attention on ERPs

N140 amplitude in the divided attention condition was intermediate between those elicited by attended and unattended stimuli during the focused attention condition. Previous studies in audition showed that the amplitude of N1 in the divided attention condition was intermediate between those elicited by attended and unattended stimuli during the focused attention condition (Hink et al., 1977, 1978; Parasuraman, 1978), which was consistent with the present results. Therefore, dividing attention between the hands may be controlled similarly to auditory divided attention, which may be explained by a capacity model of attention (Hink et al., 1977). Some researchers have also suggested that the attentional modulation of early ERP components is associated with the perceptual resource (Kok, 1997; Kida et al., 2012a,b) in the framework of multiple resources, including perceptual, central, and response resources (Wickens, 1991; Wickens and McCarley, 2008).

Regarding the distribution of attention, previous studies demonstrated a spatial gradient of attention in different modalities using visual (Mangun and Hillyard, 1988; Wijers et al., 1989; Heinze et al., 1994), auditory (Teder-Salejarvi and Hillyard, 1998; Teder-Salejarvi et al., 1999), and somatosensory ERPs (Heed and Roder, 2010), suggesting modality-independent patterns of the distribution of attention. In addition, visual and auditory ERP studies reported that attention forms a unitary zone that may expand to multiple relevant locations, but also includes the area between them (Mangun and Hillyard, 1988; Wijers et al., 1989; Heinze et al., 1994; Teder-Salejarvi et al., 1999). In contrast, a previous study on touch reported that when attention was directed simultaneously to non-adjacent fingers within one hand, ERPs in response to stimuli delivered to spatially and anatomically intervening fingers showed no attentional modulations (Eimer and Forster, 2003b). This study concluded that, in contrast to vision, the focus of somatosensory attention may be split and directed simultaneously to non-adjacent areas, thereby excluding spatially and anatomically intermediate regions from attentional processing. An MEG study also reported that somatosensory attention has a gradient and may also be divided into non-adjacent areas (Kida et al., 2018), supporting ERP results. These electrophysiological findings indicate a somatosensory-specific pattern of attention. Based on the present and previous findings, we speculate that somatosensory attention may be split between non-adjacent fingers and also between the hands. The modality specificity of the distribution of attention needs to be examined in more detail in future studies.

P300 was found for attended infrequent stimuli (targets) in both the focused and divided attention conditions, but not for identical stimuli in the control condition or unattended infrequent stimuli in the focused attention condition. This pattern is a common feature of the appearance of P300. In addition, P300 amplitude was lower when attention was directed simultaneously to both hands than when it was unilaterally focused on one hand. Previous studies reported that P300 amplitude reflects the amount of the modality-non-specific, perceptual-central resource allocated to a given task (Wickens et al., 1983; Kramer et al., 1985; Sirevaag et al., 1989; Kok, 1997, 2001; Kida et al., 2004a, 2012a,b). The gradual change observed in P300 amplitude in the present study (i.e., focused attention >divided attention >unattended or control condition) was consistent with the resource allocation view of P300 amplitude. Therefore, the decrease in P300 amplitude in the divided attention condition was assumed to be caused by the division of the modality-non-specific perceptual-central resource between the hands. The common pattern of P300 amplitude to mental counting and motor response tasks shows that the allocation of the modality-non-specific resource to both hands was independent of whether the response was covert (mental) or overt (motor).

In the divided attention condition, target probability was two-fold or target-to-target interval (TTI) was half that in the focused attention condition. P300 amplitude has been shown to decrease with high target probability and a short TTI (Kok, 2001; Polich, 2007). Therefore, it is unclear whether the decrease in P300 amplitude in the divided attention condition was due to resource division or changes in task difficulty following changes in target probability and TTI. However, the resource limitation explanation may account for the potential relationship of target probability and TTI with P300 amplitude. When target stimuli are presented more frequently (a higher target probability or shorter TTI), more resources are consumed in a given amount of time than with less frequently presented stimuli, and P300 amplitude is small. When stimuli are presented more infrequently (lower target probability or longer TTI), the structures involved in the generation of P300 may recover more fully and P300 amplitude is large (Gonsalvez and Polich, 2002; Gonsalvez et al., 2007). This resource limitation explanation accounts for the interaction between task difficulty and target stimulus probability (Kramer et al., 1986; Ruchkin et al., 1987; Polich et al., 1988). Therefore, even if the decrease in P300 amplitude by dividing attention to both hands is associated with higher target probability or shorter TTI, it may also be explained by the resource allocation view.

In contrast to the common pattern in P300 amplitude, P300 latency was longer in the divided attention condition than in the focused attention condition in the motor response task, but not in the mental counting task. This result suggests the functional dissociation of the amplitude and latency of P300. More specifically, the modality-non-specific resource may be divided between the hands without affecting the stimulus evaluation speed during the mental counting task, whereas dividing attention between the hands decreases the evaluation speed during the motor response task. It remains unclear whether motor response demands affect P300 latency, with some studies reporting no effects (Kutas et al., 1977; McCarthy and Donchin, 1981; Magliero et al., 1984; Doucet and Stelmack, 1999). In contrast, other studies noted the significant effects of motor response demands on P300 latency (Ragot and Renault, 1981; Ragot, 1984; Ragot and Renault, 1985; Pfefferbaum et al., 1986; Ragot and Lesevre, 1986; Christensen et al., 1996; Leuthold and Sommer, 1998). However, the effects of motor response demand on P300 latency have not been investigated under divided versus focused attention conditions in the somatosensory modality. In the motor response task, attention needed to be divided between the hands for both stimulus and action processes. Therefore, attentional demands required for action may be sufficiently high to decrease the stimulus evaluation speed in the motor response task to lower than that in the mental counting task with no motor response demand.

### Effects of hand and forearm postures on ERPs

Hand position, such as closely-placed hands, did not significantly affect the attentional modulation of N140 amplitude. This result shows that the attentional modulation of N140 largely depended on the anatomical space rather than the physical space. If an attentional effect on N140 amplitude is exclusively based on the physical (external) space, it is expected to disappear with closely-placed hands because spatial attention will operate equally on both to-be-attended and not-to-be-attended closely-placed hands. In contrast, if an attentional effect on N140 amplitude is based on the anatomical space, it is expected to appear in both separated-hands and closely-placed hands conditions. In addition, changes in hand position follows changes in arm posture. Therefore, a postural difference in the arms with closely-placed hands does not affect the attentional modulation of N140 amplitude.

In contrast to closely-placed hands, crossing the forearms reduced the attentional increase in N140 amplitude for standard stimuli. In a selective attention task, the ERP amplitude for standard stimuli generally reflects a pure selective attention effect, whereas that for target stimuli may contaminate a target-related effect or potential (Garcia-Larrea et al., 1995; Kida et al., 2004b, 2018). Therefore, the attentional increase in N140 amplitude in the present study represents the effects of somatosensory selective attention, which may have operated less efficiently at this stage when the forearms were crossed. Since positional and postural changes to the hands and forearms with closely-placed hands did not affect the attentional increase in N140 amplitude as discussed above, crossing the forearms may be a specific hand position and arm posture leading to changes in attentional somatosensory processing at this stage. A possible explanation for this result is based on the experimental condition that the hands and arms were placed on an unhabitual side with an unhabitual posture. This crossed-forearms condition will produce an incongruency between a representation of actual stimulus (and response) sides and a mental image of an internal space. This incongruency may result in the suppressive effect of crossing the forearms on the attentional increase in N140 amplitude. Previous studies demonstrated that attentional enhancements in early ERP amplitudes were smaller for crossed forearms than for uncrossed forearms (Eimer et al., 2001; Kennett et al., 2001; Eimer et al., 2003a; Gherri and Forster, 2012a,b), supporting the present results. Therefore, the result suggests that the attentional modulation of stimulus processing reflected by N140 amplitude is not only based on the anatomical space, but also the congruency between real and internal spaces depending on the hand position and arm posture. A psychophysical study reported that somatosensory attention was dependent on the physical space, but not on the anatomical space (Lakatos and Shepard, 1997). In contrast, ERP studies provided evidence to show that somatosensory attention was associated with an incongruency between different spatial coordinates (Eimer et al., 2001; Kennett et al., 2001). The present finding supports the latter ERP evidence. This pattern of attentional modulation associated with different spatial codes is also consistent with generators of N140, which originates from modality-specific and multisensory areas, including the second somatosensory, anterior cingulate, and prefrontal cortices (Allison et al., 1992; Tarkka et al., 1996; Waberski et al., 2002; Inui et al., 2003; Tanaka et al., 2008).

In contrast to the early N140 component, P300 amplitude was affected by both hand position and forearm posture. The behavioral measures, RT and RA, paralleled a decrease in P300 amplitude and increase in P300 latency in both the crossed-forearms and hands closely-placed conditions. P300 amplitude has been associated with equivocation or post-stimulus uncertainty (Sutton et al., 1965; Johnson, 1986; Kok, 2001) as well as resource allocation, whereas P300 latency was related to the stimulus evaluation time (Kutas et al., 1977). Therefore, crossing the forearms may decrease the resolution of post-stimulus uncertainty and increase the stimulus evaluation time through a congruency of real and learned spaces, whereas closely-placed hands exert the same effects by overlapping the attentional range at targets and non-targets.

### Effects of crossed forearms on stimulus-response coupling

Crossing the forearms significantly decreased the correlation of single-trial P300 latency with RT. As discussed above, crossing the forearms may produce an incongruency between a representation of actual stimulus (and response) sides and a mental image of a learned physical space. A previous study suggested that following accuracy maximizing instructions, subjects hesitated before pressing the button because the task used reduced their confidence of a correct response, thereby decoupling P300 latency from RT (Pfefferbaum et al., 1983). Similarly, an incongruency between actual and learned spaces may be associated with this hesitation before responding, thereby resulting in the decoupling of stimulus- and response-related processing. Crossing the forearms also decreased P300 amplitude and increased P300 latency as already discussed. Therefore, we speculate that crossing the forearms caused the decoupling of stimulus- and response-related processing, decreased the resolution of post-stimulus uncertainty, and reduced the stimulus evaluation speed concomitantly through an incongruency between real and learned spaces.

The present and previous studies using the ACF technique showed the task-dependent nature of the correlation of single-trial P300 latency with RT. A historical study on P300 latency using ACF found that speed-maximizing instructions resulted in a weaker correlation of single-trial P300 latency with RT than accuracy-maximizing instructions, suggesting the loose coupling of stimulus- and response-related processing in the former instructions and motor command output before the stimulus has been fully evaluated (Kutas et al., 1977). In contrast, another study reported the reverse effect, i.e., a weaker correlation of P300 latency with RT under accuracy-maximizing instructions than speed-maximizing instructions (Pfefferbaum et al., 1983). The latter study suggested that the difference in tasks and strategic differences for task requirements explain the discrepancies in the findings obtained as discussed above. We also previously used the ACF technique to demonstrate stronger stimulus–response coupling by the performance of a dual task than a single task (Kida et al., 2012b). Two explanations for this result, a snap decision strategy in the single-task and the lack of the resource allocated to the eliciting task during the dual-task performance, were suggested because stronger stimulus–response coupling by the dual-task performance was associated with a lower P300 amplitude and smaller number of trials with RT preceding P300 latency. However, the ratio of trials with RT preceding P300 latency was even lower in the present selective attention task (36.9–59.3%) than during single-task performance (76.8%) and was in the same range as that during the dual-task performance (53.7%). This is because the present study employed a type of selective attention task where somatosensory stimuli were presented to many fingers in bilateral hands and subjects had to discriminate one or two target stimuli, i.e., task demand was higher than the simple somatosensory oddball task used in the previous study where two types of somatosensory stimuli were presented to unilateral fingers. RT was earlier in the previous study (349.5 and 336.6 ms in experiments 1 and 2, respectively) than in the present study (ranging between 363.5 and 394.7 ms depending on the task condition, Tables 2, 5), supporting the selective attention task used herein being a more demanding task than the oddball task used in the previous study. Therefore, the snap decision is not the main cause for the changes observed in the coupling of stimulus- and response-related processing in the present study. Another suggestion, the lack of the perceptual-central resource, is also not straightforward to explain our previous findings and the present results because the two studies showed the reverse pattern for P300 amplitude and the correlation of single-trial P300 latency with RT. Therefore, the present and previous studies showed the importance of task features in interpreting the correlation of single-trial P300 latency with RT.

### Hand preference of the P300 latency-RT correlation

In the present study, the correlation between single-trial P300 latency and RT was stronger for the right hand than for the left hand in right-handed subjects. Previous studies reported a hand preference (i.e., a difference between the dominant and non-dominant hands) in motor tasks and sensorimotor tasks (Beste et al., 2009), whereas others showed no hand preference in a tactile perceptual task (Finlayson and Reitan, 1976). Regarding neural activation, movements with the non-dominant hand were associated with stronger and more extended brain activation than those with the dominant hand (Leocani et al., 2001; Potgieser et al., 2015). In contrast, an ERP study found that theta power related to handwriting was higher with the dominant hand than with the non-dominant hand (Pei et al., 2021). In addition to these findings from psychophysics and neuroimaging, the present study provides evidence for a hand preference for stimulus–response coupling using the combined measure of behavioral and neural response times. This hand preference of stimulus–response coupling was eliminated by crossing the forearms, but not by placing the hands close together or dividing attention between the hands. As already discussed, crossing the forearms may produce an incongruency between real (external) and learned (internal) spaces, which may eliminate the hand preference in the stimulus–response translation process.

### Somatosensory mismatch responses

In the present study, N140 amplitude was higher for deviant stimuli than for standard stimuli in all conditions, including attended and unattended stimuli. This amplitude increase was associated with somatosensory mismatch negativity (s-MMN), which has been reported to occur in the range of this latency in passive tasks (Kekoni et al., 1997; Kida et al., 2004c; Restuccia et al., 2007, 2009; Chen et al., 2014; Shen et al., 2018a; He et al., 2020). MMN is considered to reflect the automatic detection of stimulus changes in the sensory environment (Naatanen, 1992; Naatanen et al., 2007) and has recently been associated with predictive coding (Kimura, 2012; Stefanics et al., 2014). The first demonstration of MMN was in both the unattended and attended channels in an auditory selective attention task (Naatanen et al., 1978), suggesting the automatic nature of MMN. However, s-MMN was previously detected in passive tasks, such as reading (Kekoni et al., 1997; Restuccia et al., 2007, 2009) and video watching (Chen et al., 2014; Shen et al., 2018b), both of which are general MMN recording procedures. In contrast, the present study observed s-MMN in both attended and unattended deviant stimuli in a selective attention task, suggesting that the generation of the response is independent of attention. Similar attention-independent responses to stimulus onset, offset, and change have been demonstrated in both active and passive tasks (Yamashiro et al., 2008, 2009). Previous studies found s-MMN in frontal (Kekoni et al., 1997; Kida et al., 2004c) and parieto-occipital sites (Restuccia et al., 2007, 2009). We also detected s-MMN in frontal and central sites, supporting previous ERP findings. Placing the hands close together and crossing the forearms had no impact on the higher amplitude for deviant stimuli than for standard stimuli, thereby supporting the primarily automatic nature of MMN.

### Limitations

Since we recorded ERPs from a limited number of electrodes, we were unable to perform a source-level analysis or current source density analysis. However, N140 and P300 have both been extensively examined and an abundant amount of information has been obtained on their generators (Allison et al., 1992; Tarkka et al., 1996; Valeriani et al., 2000, 2001; Tanaka et al., 2008). Therefore, it is possible to speculate about the brain regions involved in the modulation of ERP components. Furthermore, we did not perform the divided attention condition when the hands were closely located or when the forearms were crossed. These conditions may provide insights into the interaction between different spatial coordinates in somatosensory attention. Another limitation is the small sample size; therefore, we performed a post-hoc power analysis (Supplementary Material). The results obtained showed that most of the significant differences observed in ANOVAs, MANOVAs, and *t*-tests remained and, thus, our suggestions are effective. However, some analyzes showed low statistical power, suggesting higher type II error in some results, which was at least partly due to the small sample size. Therefore, further studies are warranted.

## Conclusion

In conclusion, the present study demonstrated that the pattern of the somatosensory-attention effect on ERPs during focused and divided attention was similar to that in vision and audition. Therefore, somatosensory attention may be split between the hands, but follows some delay in modality-non-specific late processing by dividing resources between the hands depending on task demands (mental or motor). The present results in the somatosensory modality support the classical resource allocation view of the P300 amplitude in both motor response and mental tasks and also provides additional evidence for s-MMN. The effect of somatosensory-spatial attention reflected by N140 amplitude may be affected by crossed forearms, but not by closely-placed hands, whereas modality-non-specific late processing interfered uniformly with both. A combined measure of neural (P300) and behavioral (RT) response times revealed specific changes in stimulus–response coupling and hand preference. Therefore, hand position and arm posture differently affected the attentional modulation of somatosensory processing at different stages as well as stimulus-response coupling.

## Data availability statement

The original contributions presented in the study are included in the article/Supplementary material, further inquiries can be directed to the corresponding author.

## Ethics statement

The studies involving humans were approved by Ethics Committee, Graduate School of Comprehensive Human Sciences at the University of Tsukuba. The studies were conducted in accordance with the local legislation and institutional requirements. The participants provided their written informed consent to participate in this study.

## Author contributions

TKi, TKa, and YN contributed to conception and design of the study. TKi and TKa performed the experiment and analysis. TKi wrote the first draft of the manuscript. All authors contributed to the article and approved the submitted version.
