# Supplementary material for: ERP evidence of attentional somatosensory processing and stimulus-response coupling under different hand and arm postures
Source: Front Hum Neurosci. 2023 Nov 1;17:1252686. doi: 10.3389/fnhum.2023.1252686 (PMC10676239; doi:10.3389/fnhum.2023.1252686)
Supplement: Supplementary file 1 [file Table_1.DOCX]

**ERP evidence of attentional somatosensory processing and stimulus-response coupling under different hand and arm postures**

Tetsuo Kida, Takeshi Kaneda, Yoshiaki Nishihira

**Supplementary Material**

The results of a post-hoc power analysis associated with repeated measures ANOVAs and paired *t*-tests performed in the present study are shown. This analysis was conducted using G*Power software (Ver. 3.1.9.7). The error probability α was set to 0.05. The results obtained were only presented when ANOVAs or *t*-tests showed significant effects. Power is expressed in (1-β error probability). Cohen’s f(U) in ANOVAs or dz in *t*-tests are presented as effect size measures. Details (e.g., levels) in ANOVAs are described in the Materials and Methods section. Cc, Central electrode contralateral to the stimulus hand; amp, amplitude; lat, latency, RT, reaction time; RA, response accuracy

| Behavioral or  ERP measures | Test type | Factor (no. of levels) | f(U) or dz | Power  (1-β) |
| --- | --- | --- | --- | --- |
| Exp.1: Focused vs. divided attention | | | | |
| N140 amp (Fz) | 2-way ANOVA | Attention (4) | 1.49 | 0.99 |
|  |  | Stimulus type (2) | 1.49 | 0.97 |
| N140 amp (Cc) | 2-way ANOVA | Attention (4) | 1.04 | 0.99 |
|  |  | Stimulus type (2) | 1.22 | 0.90 |
| P300 amp (Pz) | *t*-test | Divided vs. focused | 1.31 | 0.98 |
|  |  |  |  |  |
| Exp.2: Focused vs divided attention | | | | |
| RT | 2-way ANOVA | Attention (3) | 0.72 | 0.71 |
| RA | 2-way ANOVA | Attention (3) | 0.90 | 0.89 |
| N140 amp (Fz) | 2-way ANOVA | Attention (4) | 1.49 | 0.99 |
|  |  | Stimulus type (2) | 0.94 | 0.71 |
| N140 amp (Cc) | 2-way ANOVA | Attention (4) | 1.12 | 0.99 |
|  |  | Stimulus type (2) | 1.25 | 0.91 |
|  |  | 2-way interaction | 0.61 | 0.69 |
| P300 amp (Pz) | *t*-test | Divided vs. focused | 1.31 | 0.96 |
| P300 lat (Pz) | *t*-test | Divided vs. focused | 0.36 | 0.17 |
|  |  |  |  |  |
| Exp.2: Closely-placed vs. separated hands | | | | |
| N140 amp (Fz) | 3-way ANOVA | Attention (2) | 1.88 | 0.99 |
|  |  | Stimulus type (2) | 1.12 | 0.85 |
| N140 amp (Cc) | 3-way ANOVA | Attention (2) | 1.30 | 0.93 |
|  |  | Stimulus type (2) | 1.49 | 0.98 |
|  |  | Hand position (2) | 0.94 | 0.71 |
| P300 amp (Pz) | *t*-test | Close vs. separated | 0.74 | 0.55 |
| P300 lat (Pz) | *t*-test | Close vs. separated | 0.77 | 0.58 |
|  |  |  |  |  |
| Exp.3: Crossed vs. uncrossed forearms | | | | |
| RT | 2-way ANOVA | Forearm posture (2) | 0.72 | 0.49 |
| RA | 2-way ANOVA | Forearm posture (2) | 1.42 | 0.97 |
| N140 amp (Fz) | 3-way ANOVA | Attention (2) | 1.56 | 0.99 |
|  |  | Stimulus type (2) | 1.15 | 0.87 |
| N140 amp (Cc) | 3-way ANOVA | Attention (2) | 1.42 | 0.97 |
|  |  | Stimulus type (2) | 1.49 | 0.99 |
| P300 amp (Pz) | *t*-test | Crossed vs. uncrossed | 0.82 | 0.64 |
| P300 lat (Pz) | *t*-test | Crossed vs. uncrossed | 1.11 | 0.88 |
|  |  |  |  |  |
| Exp.3: Simple effect of attention in MANOVA | | | | |
| ERP measure | Stimulus type | Forearm posture | f(U) | Power  (1-β) |
| N140 amp (Fz) | Standard | Uncrossed | 1.49 | 0.99 |
|  |  | Crossed | 1.11 | 0.99 |
|  | Deviant | Uncrossed | 0.92 | 0.96 |
|  |  | Crossed | 0.89 | 0.94 |
